# Increasing Human Papillomavirus Vaccination in a Federally Qualified Health Center Organization Using a Systems-Based Intervention Integrating EHR and Statewide Immunization Information System

**DOI:** 10.1007/s10900-021-00965-6

**Published:** 2021-07-31

**Authors:** Debra M. Vinci, Jessica Ryan, Maureen Howard, Dallas Snider, Brandy Strahan, Gregg Smith, Rebecca McClain

**Affiliations:** 1 Department of Movement Sciences & Health, University of West Florida, 11000 University Parkway, Pensacola, FL 32514 USA; 2 Department of Health Sciences & Administration, University of West Florida, 11000 University Parkway, Pensacola, FL 32514 USA; 3 Department of Information Technology, University of West Florida, 11000 University Parkway, Pensacola, FL 32514 USA; 4 School of Nursing, University of West Florida, 11000 University Parkway, Pensacola, FL 32514 USA; 5 Tallahassee, FL USA

**Keywords:** HPV, Information immunization system (IIS), Systems change, Vaccination rates

## Abstract

Public acceptance of the HPV vaccine has not matched that of other common adolescent vaccines, and HPV vaccination rates remain below the Healthy People 2020 target of 80% compliance. The purpose of this study was to evaluate the capacity of nine pediatric clinics in a Federally Qualified Health Center organization to implement a systems-based intervention targeting office staff and providers using EHRs and a statewide immunization information system to increase HPV vaccination rates in girls and boys, ages 11 to 16 over a 16-month period. System changes included automated HPV prompts to staff, postcard reminders to parents when youths turned 11 or 12 years old, and monthly assessment of provider vaccination rates.

During the intervention, 8960 patients (11–16 yo) were followed, with 48.8% girls (n = 4370) and 51.2% boys (n = 4590). For this study period, 80.5% of total patients received the first dose of the HPV vaccine and 47% received the second dose. For the first dose, 55.5% of 11 year old girls and 54.3% of 11 year old boys were vaccinated. For ages 12 to 16, first dose vaccination rates ranged from the lowest rate of 84.5% for 14 yo girls up to the highest rate of 90.5% for 13 yo boys. Logistic regression showed age was highly significantly associated with first dose completion (OR 1.565, 95% CI 1.501, 1.631) while males did not have a significant association with first dose completion compared to females. The intervention increased overall counts of first and second HPV vaccination rates.

## Introduction

Over 34,000 cases of cancer are caused by the human papillomavirus (HPV) each year in the United States [1]. Cancers originating from HPV include cancers of the cervix, vagina, vulva, penis, anus, and oropharynx [1]. The first HPV vaccine was licensed in 2006 and subsequent versions became available in 2009 and 2014 [2].

Current CDC recommendations for HPV vaccination are determined by the patient’s age when the initial HPV vaccine dose is administered. For adolescents starting the vaccination series between the ages of 9 and 14 years, two doses separated by 6–12 months are sufficient. For adolescents beginning the HPV vaccination series at 15 years of age or older, three doses are recommended. The second dose should be administered one to two months after the first dose and the third dose should be administered six months after the first dose [3].

Acceptance of the HPV vaccine has not matched that of other common adolescent vaccines, and HPV vaccination rates remain below the Healthy People 2020 target of 80% compliance [4]. According to 2018 data, 68% of US adolescents aged 13–17 years had received the first HPV vaccine dose, but only 51% of US adolescents had completed the HPV series [5]. Many factors contribute to low HPV vaccination compliance rates. Opportunities for HPV vaccination are missed because many adolescents do not attend routine primary or preventive healthcare visits [6]. Parents may refuse HPV vaccination for their children due to cost, lack of accurate information about the vaccine’s benefits, low perceived risk of HPV infection, and/or concerns about the vaccine’s effect on adolescent sexual behavior [7]. Providers may fail to communicate strong and consistent recommendations for adolescent patients to receive HPV vaccinations [8, 9]. Additionally, there is no broad mandate for HPV vaccination of school-aged adolescents, as HPV vaccination is required for school attendance in only three states [10]

In 2014, increasing the rates of HPV vaccination was recognized as a public health priority and three related goals were identified: (1) improve clinical methods to maximize the recommendation and administration of the HPV vaccine; (2) increase acceptance of HPV vaccination; and (3) improve HPV vaccine availability [11]. Evidenced-based clinical strategies to increase HPV vaccination rates have been achieved through systems-based interventions including provider and staff training on vaccination policy and procedures, unified provider communication approach on vaccination, use of Electronic Health Records (EHR), patient reminders, recall notices, and automated record alerts when patients are due for vaccination [12–14]. Additionally, statewide immunization information systems (IISs) provide healthcare providers with a tool to track immunization records of all ages, including HPV vaccination rates [15].

Preliminary research has found promising results using systems-based interventions in a variety of settings including urban hospital primary care clinics; urban private pediatric practices; urban and suburban clinics associated with academic hospitals; urban and suburban primary care clinics; and Federally Qualified Health Centers (FQHCs), with the majority of interventions targeting girls [16]. The aim of this study was to evaluate the capacity of a FQHC organization to implement a systems-based intervention targeting office staff and providers using EHRs and a statewide IIS to increase vaccination rates in girls and boys, ages 11 to 16 over a 16-month time period.

## Methods

### Study Setting

This is a systems-based intervention to increase HPV vaccination in boys and girls in a FQHC organization with 30 clinic offices providing medical and dental services to patients in Charlotte, Hendry, and Lee Counties in southwest (SW) Florida. Nine pediatric clinics participated in this intervention. Funding for this project was from the Florida Comprehensive Cancer Control Program (FCCCP) within the Florida Department of Health (FDOH). FDOH acknowledges and values the continued improvement of HPV vaccination rates and is committed to enhanced collaboration to implement sustainable health system interventions [17]. This study was reviewed and approved by the Ethics and Human Research Protection Program at the Florida Department of Health, Department of Health Ethics and Human Research Protection Program, and the University of West Florida (UWF) Institutional Review Board.

### Subjects

Using de-identified data submitted by nine participating pediatric clinics, HPV vaccination rates were assessed in girls and boys, aged 11 to 16, from March 2013 through December 2019. The 16- month time period of interest was September 2018 to December 2019, which is the time period after the systems-based intervention went into effect.

### Intervention

The FDOH was interested in developing partnerships with FQHCs to support systems-based interventions to increase HPV vaccinations. Solicitations for intervention studies to implement systems-based interventions were released in Fall 2017 with a FQHC in SW Florida selected for this intervention in February 2018.

## Procedures

### System Changes

In July 2018, this SW Florida FQHC’s Quality Assurance and Quality Improvement (QA/QI) Team for the nine participating pediatric clinics implemented policy and procedural changes to improve the use of the organization’s IIS. The changes included the use of patients’ EHR and Florida State Health Online Tracking System (SHOTS). Florida SHOTS is an IIS in which immunizations administered by participating providers in Florida are tracked. It is a free, statewide, centralized immunization registry that can be accessed by enrolled healthcare providers, schools, and parents [18].

The policy and procedural changes included: (1) provider reminders to alert staff through automated prompts on patient forms and within the EHR system; (2) patient reminders to alert parents/caregivers of patients due for vaccination on their 11th and 12th birthdays through postcard mailings; and (3) provider assessment and feedback through verbal and written notification of individual physician vaccination rates for overall patient population at monthly clinical meetings. By integrating Florida SHOTS into the organization’s policies and procedures, patients who have received vaccinations at clinics outside the FQHC system can be updated into the patient’s EHR. Also, the FQHC implemented an Incentive Program for Adolescent Immunization compliance for pediatric providers and staff starting in January 2018, seven months prior to the implementation of this systems-based intervention.

### Training

In August 2018, the FQHC’s QA/QI Team implemented policy and procedural changes. The training was developed and presented by the QA/QI Director. Thirty-five training sessions were held and 199 medical assistants and office staff members were trained on best practices and new policies and procedures for increasing HPV vaccination rates during August 2018. Separate training sessions were held for the 46 providers (ARNP, DO, MD, PA) at their monthly meetings from August 2018 to December 2019. Each month (September 2018–December 2019) the QA/QI Team went to each of the separate clinic offices to address barriers the clinics were facing, review HPV vaccination data, and train any new staff.

### HPV Vaccination Intervention

**Fig. 1:**
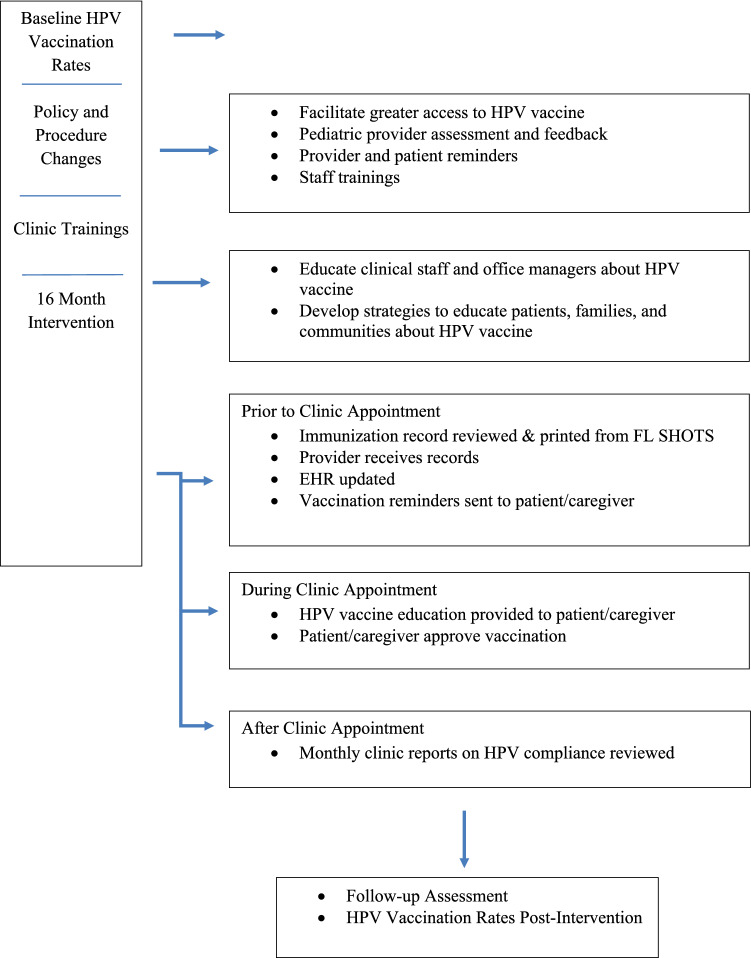
The HPV vaccination intervention process at each of the nine pediatric clinics

The HPV vaccination protocol was implemented in nine pediatric clinics and involved the use of FQHC’s EHR and the FDOH statewide immunization registry, Florida SHOTS. Each month, clinic site managers reviewed patients using Florida SHOTS to determine patients who were due for vaccinations including HPV. Figure 1 describes the HPV Vaccination Intervention process that took place at each of the nine pediatric clinics.

### Patient Appointment

Prior to a clinic appointment, the clinic site manager would print patients’ HPV immunization records from Florida SHOTS, highlighting which patients were due for their HPV vaccination and give the record to the provider for review. Patient reminders were sent to the patient/parent/caregiver via postcard and/or phone call, depending on patients’ preference. Additionally, all pediatric clinics would offer “vaccine only” appointments to decrease wait times for patients.

During the patient appointment, HPV vaccination education was provided to the patient/parent/caregiver using the “announcement approach” [12] where the HPV vaccination was viewed as a part of the routine vaccinations for this age group. HPV vaccination education was given verbally along with written information in triage, during the patient visit, and patient’s time with the provider. The clinics have bilingual staff for Spanish-speaking patients as well as a Language Assistance Line to provide interpretation if needed. The HPV educational activities were documented in the EHR.

At post-visit, records in Florida SHOTS were updated and printed, highlighting upcoming vaccines and information was added to the recall list. Each month, the organization's Management Information System (MIS) generated monthly reports on HPV compliance that included non-compliant patients (11–15 years old), where the youth were in the HPV vaccination series (Never, Follow-Up, Completed), follow-up attempts on the type of reminder sent, and date of first and second follow-up calls. Data from monthly reports were shared with pediatric clinic sites.

## Data Measures and Statistical Analysis

Using a non-randomized design, de-identified individual-level HPV vaccination data were collected from the pediatric clinics using an approved HPV Intervention Database. Total observations were 8,960. Patients who received the first dose within 12 months of the study’s end date but had not received the second dose yet were not considered non-compliant. The data were then analyzed for descriptive and inferential statistics using Microsoft Excel and SAS 9.4 for Windows.

The descriptive data of patients ages 11 to 16 years receiving HPV vaccination first and/or second dose was calculated at baseline prior to the intervention and again during the 16-month period after the intervention. A logistic regression model was analyzed with first dose completion as the dependent variable. The independent variables were age and sex. Ethnicity was not considered since this variable was not in the HPV Intervention Database. Pearson’s chi-squared tests were performed on the baseline versus intervention group for first dose completion as well as second dose completion. For the chi-squared test on first dose completion, the baseline group included all patients in the study population who had turned 11 years old before September 1, 2018. Patients who received the first dose of the HPV vaccination were then removed from the dataset and the remaining patients were considered the intervention group. For the chi-squared test on second dose completion, all patients who had not received the first dose of the HPV vaccination were removed from the dataset. Patients were included in the baseline group if they had received their first dose prior to September 1, 2017 and, therefore, were eligible to receive their second dose by September 1, 2018. Patients were included in the intervention group if they had not received their second dose before September 1, 2018 and received their first dose before December 31, 2018.

## Results

**Fig. 2:**
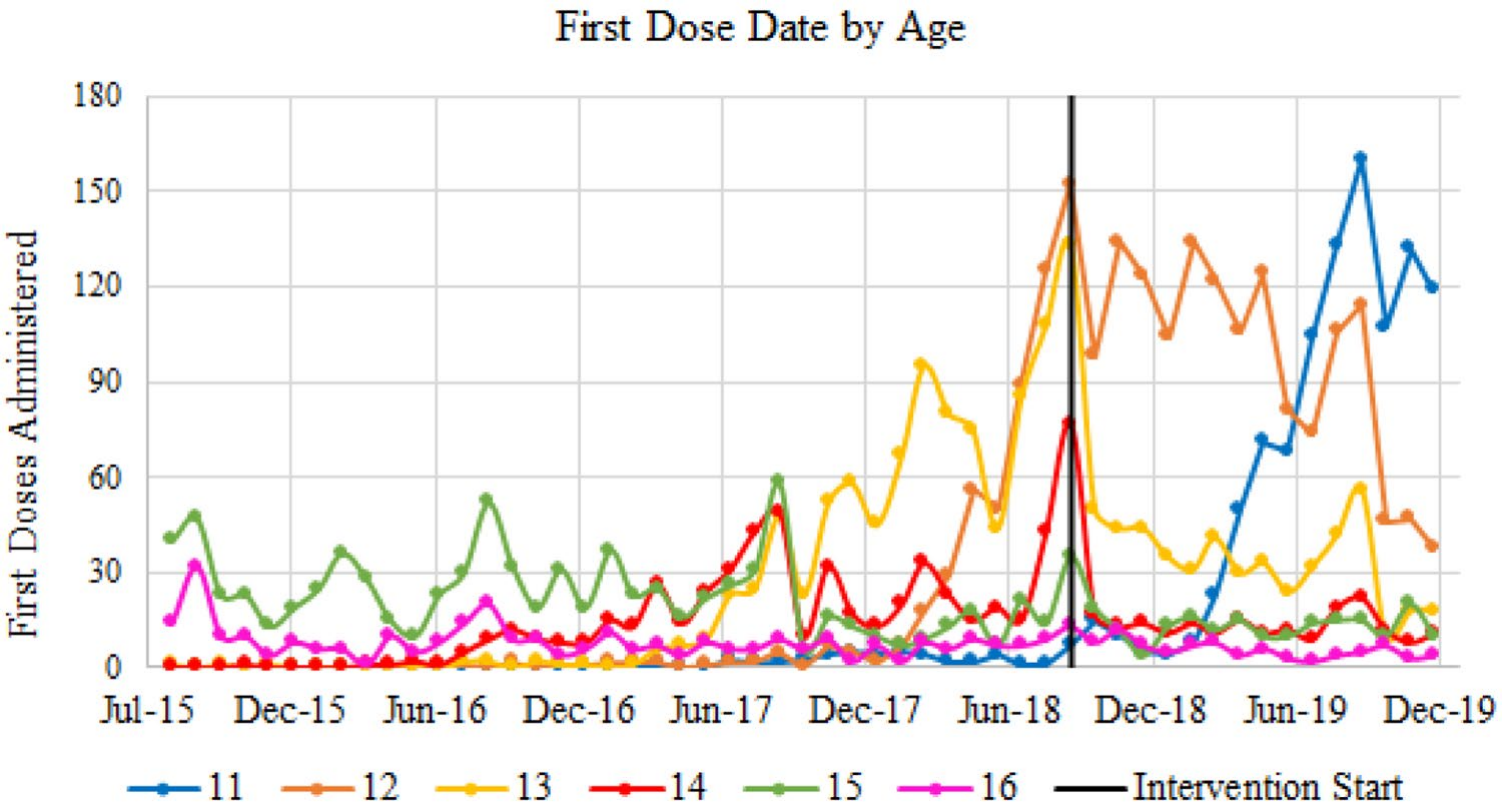
First dose by age prior to the intervention (January 2018–July 2018) and during the Intervention (August 2018 to December 2019)

There were 8960 patients (11–16 years old) followed during the nearly 7 year study period with 48.8% girls (n = 4370) and 51.2% boys (n = 4590). For this study period, 80.5% of FQHC patients received the first HPV vaccine dose and 47% received the second dose. For the first dose, 55.5% of 11 year old girls and 54.3% of 11 year old boys were vaccinated. From age 12 to age 16, first dose vaccination rates ranged from the lowest rate of 84.5% for 14 yo girls up to the highest rate of 90.5% for 13 yo boys (Table 1). Figure 2 presents the first dose by age prior to the intervention (January 2018–July 2018) and during the intervention (August 2018 to December 2019).

**Table 1:** First and second dose compliance rates by gender (N = 8960)

Age	Total	Count		Percentages	
		First shot	Second shot	First shot	Second shot^a^ (%)
Girls (y.o.)					
11	1058	587	78	55.5	13.3
12	1135	1012	539	89.2	53.3
13	790	709	507	89.7	71.2
14	472	399	280	84.5	70.2
15	638	564	470	88.4	83.3
16	277	243	208	87.7	85.5
Total	4370				
Boys (y.o.)					
11	1086	590	82	54.3	13.8
12	1162	1022	525	88.0	51.4
13	886	802	551	90.5	68.7
14	469	410	255	87.4	62.3
15	678	605	504	89.2	83.3
16	309	269	219	87.1	81.4
Total	4590				
Total	8960	7203 (80.49%)	4218 (47%)		

^a^Based on patients who completed first dose HPV vaccination

**Fig. 3:**
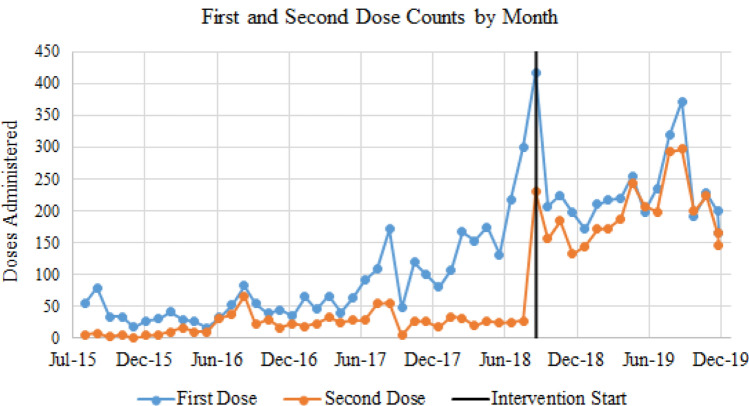
Count of first and second HPV dose by month from July 2015) when the intervention started (July 2018) and 16-month intervention period (September 2018–December 2019)

**Fig. 4:**
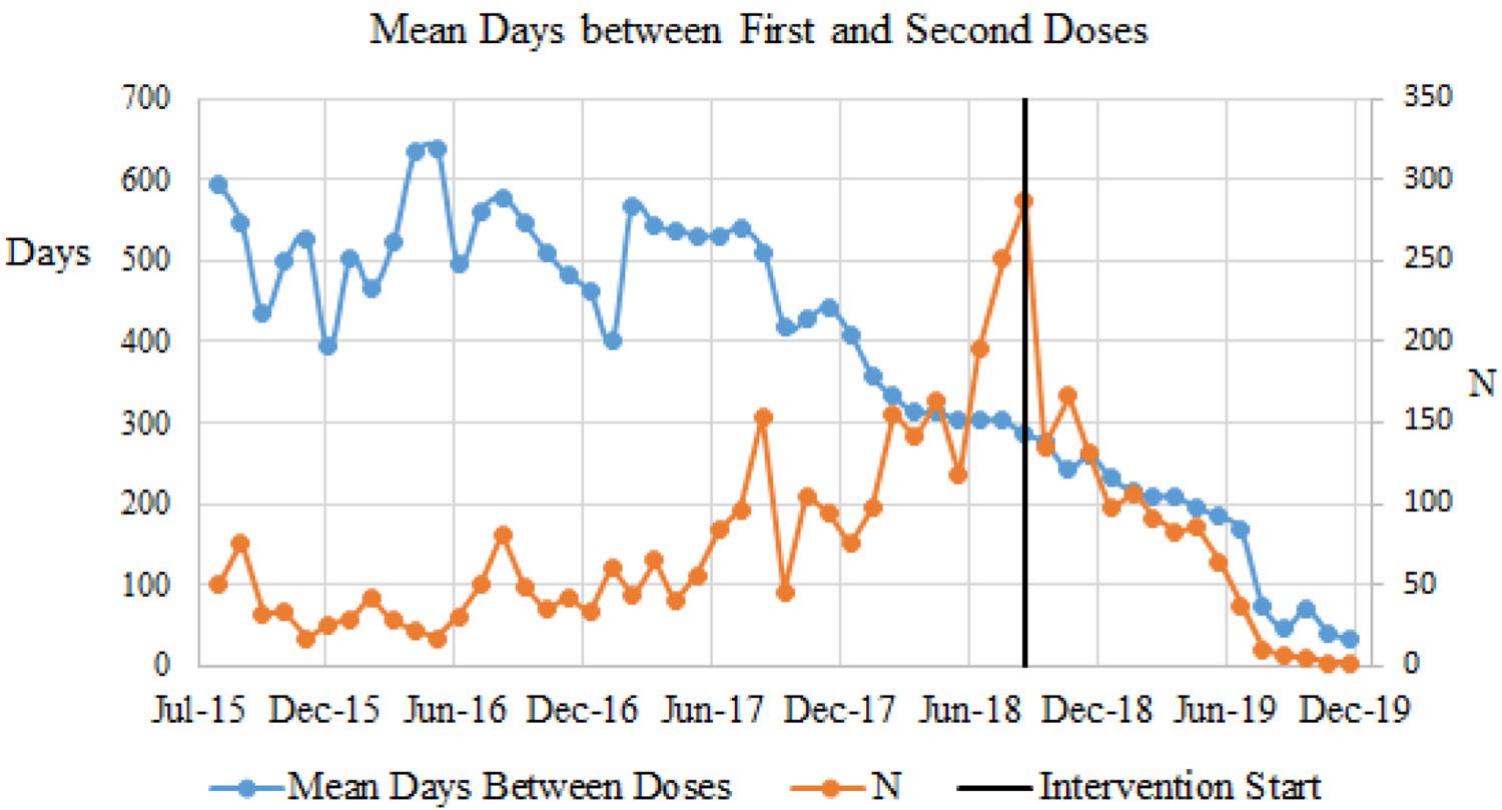
Mean days between first and second doses by month from July 2015, when the intervention started (July 2018), and 16-month intervention period (September 2018–December 2019). The decline in second dose vaccinations after the start of the intervention is a result of the limitations of the dataset to a 16-month time period after the intervention

For the second HPV dose, the lowest age group was 11 yo (13.3% girls; 13.8% boys) with compliance rates increasing with age (Table 1). Figure 3 illustrates the HPV first and second dose by month from July 2015; when the intervention started (July 2018) and 16-month intervention period (September 2018–December 2019). For this same time period, mean days between first and second doses are displayed in Fig. 4. Logistic regression (Table 2) showed age was highly significantly associated with first dose completion (OR 1.565, 95% CI 1.501, 1.631) while males did not have a significant association with first dose completion compared to females.

**Table 2:** Logistic regression model of first dose completion (2013–2019)

	Maximum likelihood estimates	Standard error	Wald chi-square	P > ChiSq	Odds ratio estimates	95% wald confidence limits	
Intercept	− 4.196	0.261	258.58	< 0.0001			
Age	0.448	0.021	450.93	< 0.0001	1.565	1.501	1.631
Male	− 0.001	0.055	0.0005	0.983	0.999	0.897	1.112

In the baseline group, 60.08% of patients received the first dose of the HPV vaccination. After the intervention, 67.42% of patients received the first dose. Patients were more likely to receive a first dose of the HPV vaccination after the initiative χ^2^ (1, 11,191) = 64.94, p < 0.00001 (Table 3). Patients in the baseline group who did not receive the first dose were then added to the intervention group which explains why N = 11,191 is greater than the study population.

**Table 3:** Contingency table of first dose completion for baseline and intervention groups

Initiative					
	Baseline	Intervention	Total	χ^2^	p-value
1st Dose					
Received					
Count	3504	3613	7117	64.94	p < 0.00001
% within 1st dose	49.23%	50.77%	100.00%		
% within initiative	60.08%	67.42%	63.60%		
% of total	31.31%	32.28%	63.60%		
Not received					
Count	2328	1746	4074		
% within 1st dose	57.14%	42.86%	100.00%		
% within initiative	39.92%	32.58%	36.40%		
% of total	20.80%	15.60%	36.40%		
Total					
Count	5832	5359	11,191		
% within 1st dose	52.11%	47.89%	100.00%		
% within initiative	100.00%	100.00%	100.00%		
% of total	52.11%	47.89%	100.00%		

Counts of youth who received the second dose of the HPV vaccine rose after the intervention (1470 before and 2629 after). However, counts for youth who did not receive the second dose also rose (111 before versus 670 after) leading to a 92.98% rate for patients receiving the second dose before and a 79.69% rate for patients receiving the second dose after the intervention (Table 4). Patients were less likely to receive the second dose of the HPV vaccination after the initiative χ^2^ (1, 4880) = 140.39, p < 0.00001.

**Table 4:** Contingency Table of Second Dose Completion for Baseline and Intervention Groups

Initiative					
	Baseline	Intervention	Total	χ^2^	p-value
2nd dose					
Received					
Count	1470	2629	4099	140.39	p < 0.00001
% within 2nd dose	35.86%	64.14%	100.00%		
% within initiative	92.98%	79.69%	84.00%		
% of total	30.12%	53.87%	84.00%		
Not received					
Count	111	670	781		
% within 2nd dose	14.21%	85.79%	100.00%		
% within initiative	7.02%	20.31%	16.00%		
% of total	2.27%	13.73%	16.00%		
Total					
Count	1581	3299	4880		
% within 2nd dose	32.40%	67.60%	100.00%		
% within initiative	100.00%	100.00%	100.00%		
% of total	32.40%	67.60%	100.00%		

The service area for the FQHC organization included nine pediatric clinics in the Cape Coral-Fort Myers MSA metro area [19]. Overall, 80% (7212 patients) received the first HPV vaccine dose with 58% returning for the second dose (4218 patients). Less than 1% received the third dose. Table 5 summarizes the first dose compliance for the nine clinics with an overall average for the FQHC study population of 80.49%. Five clinics had compliance rates over 82% and the remaining four clinics between 72 and 79.85%. This represents an increase from pre-intervention compliance rates of 64.94% in 2017 (Table 6).

**Table 5:** First dose compliance rates by clinics (2018–2019)

Service center	Total patients	First dose	Compliance (%)
Clinic #1	792	663	83.71
Clinic #2	822	592	72.02
Clinic #3	680	579	85.15
Clinic #4	1246	1027	82.42
Clinic #5	1811	1519	83.88
Clinic #6	839	669	79.74
Clinic #7	773	640	82.79
Clinic #8	1057	844	79.85
Clinic #9	940	679	72.23
Grand total	8960	7212	80.49^a^

^a^Based on Grand Total; Average % compliance by Clinic lower related to rounding numbers

**Table 6:** first dose compliance rates by clinics (2017, Pre-intervention

Service center	Total patients	First dose	Compliance (%)
Clinic #1	357	246	68.91
Clinic #2	102	44	43.14
Clinic #3	320	222	69.38
Clinic #4	767	448	58.41
Clinic #5	894	633	70.81
Clinic #6	396	258	65.15
Clinic #7	247	153	61.94
Clinic #8	504	337	66.87
Clinic #9	318	195	61.32
Grand total	3905	2536	64.94^a^

^a^Based on Grand Total; Average % compliance by Clinic lower related to rounding numbers

## Discussion

A systems-based HPV intervention was implemented in a FQHC organization to determine if they had the capacity to increase vaccination rates in girls and boys, ages 11 to 16, over a sixteen-month time period. FQHC established policies and procedures that included the following evidenced-based practices to increase HPV vaccination rates: provider and staff education on vaccination policy and procedures; use of EHR, patient reminders, recall notices, and automated record alerts when patients were due for vaccination [12–14]; and parent education announcing vaccinations due, including the HPV vaccine [12]. Overall, the results indicated that the FQHC organization had the capacity to implement a systems-based intervention with patients more likely to receive the first dose of the HPV vaccination after the intervention. Additionally, nine pediatric clinics reported 80.49% of patients (11–16 yo) received the first dose of the HPV vaccine during the study period (Table 1). This meets the Healthy People 2020 [4] target of 80% and is higher than the 2018 National Immunization Survey with HPV vaccination rates of 68.1% for ≥ 1 dose of HPV vaccine (13–17 years) [5] and data for Florida at 59.8% [20].

Notably, the FQHC organization utilized their EHR to provide patient reminders. They also integrated Florida SHOTS, the statewide IIS, into their immunization procedures to determine if patients had HPV immunizations from other providers outside the organization. IISs are intended to facilitate cooperation between public health agencies and vaccination providers such as healthcare systems, practitioners, schools, and health departments [15]. Computerized IIS databases provide a platform to collect and consolidate records of vaccinations administered by participating providers within a designated geographical area [15]. To date, most of these IISs are not mandated and rely on voluntary state participation, which can impact the accuracy of immunization reporting. By combining Florida SHOTS with EHR, FQHC pediatric offices were able to better assess vaccination needs of their patients and increase the first dose of the HPV vaccination, especially in girls.

While the intervention did increase patients receiving the first dose of the vaccine (pre = 60%; post = 67.42%), the second dose rate decreased after the intervention. There are limited studies that provide insight into decreased adherence to the second dose [21]; however, from the data a time component factor is clear. There are nearly 7 years of pre-intervention data, yet only 16 months of data available post-intervention. A reason for a decrease in HPV second dose can be related to competing priorities within a clinic setting, low health literacy, cultural/language barriers, and vaccine stigma [22]; communication of the importance of HPV in preventing cancer [20] intervals recommended between doses that extended out beyond the intervention timeline [2] and potential of providers extending the follow-up vaccination to 1-year following pediatric visit (J. Ryan, personal communication, January 12, 2020). It is important to note that the mean days between the first and second doses did decrease with the in interventions as illustrated in Figs. 3 and 4. Simply put, the longer time goes on, the more likely that the youth will eventually receive the second dose of the HPV vaccine.

This study also supports the importance of FQHCs’ role in providing access to the HPV vaccinations for the uninsured with only 5.5% of the organization’s patients having private insurance. To effectively increase HPV vaccination rates, theory-informed interventions and implementation strategies are urgently needed [23]. The FQHC HPV vaccination intervention aligns quite well with the framework of the P3 Model [24] which combines key elements of several standard health promotion and behavioral models. The P3 Model was developed to guide the collaboration that must take place between the practice, provider, and patient in order to improve preventative healthcare. The FQHC intervention provides an example of how the P3 Model could provide a framework for coordinating the roles of all three levels of the clinical encounter.

During the FQHC intervention, the practice level was responsible for collecting HPV vaccination data from Florida SHOTS and integrating them into the EHR system, sending reminders to providers when patients were due for vaccination, and conducting staff training sessions on policies and procedures to increase HPV vaccination. The provider level was responsible for sending vaccination reminders to patients and setting follow-up appointments, establishing a standard recommendation for HPV vaccination, providing examples of language to be used to answer frequently asked questions, and ensuring the ability to serve all patient populations (e.g. Spanish-speaking staff). The patient level was responsible for taking advantage of community and clinic materials (e.g. pamphlets, magazines, signs, videos) to become educated about HPV vaccination benefits and protocols and to initiate and complete the HPV vaccination series. Using this model in future interventions could provide a framework for program implementation and evaluation.

## Limitations

There are limitations to this study. The intervention was limited to one FQHC in SW Florida which may not be representative of other primary care pediatricians in other FQHCs or in private practice. Additionally, this study used a non-randomized design so there was no control group for comparison. We were also not able to address the impact of ethnicity on initiation and completion of HPV vaccination series since this information was not captured in the database. Finally, there are unknowns related to the impact of the organization’s Vaccination Incentive Program, staff training related to new employees hired after August 2018, and accuracy of contact information for patients to initiate HPV vaccination and follow-up on receiving the second HPV vaccination.

## Data Availability

Dataset available on request. Custom code is available upon request.
